# A Software Tool to Model Genetic Regulatory Networks. Applications to the Modeling of Threshold Phenomena and of Spatial Patterning in *Drosophila*

**DOI:** 10.1371/journal.pone.0010743

**Published:** 2010-05-27

**Authors:** Rui Dilão, Daniele Muraro

**Affiliations:** Nonlinear Dynamics Group, Instituto Superior Técnico, Lisbon, Portugal; Centre for Genomic Regulation (CRG), Universitat Pompeu Fabra, Spain

## Abstract

We present a general methodology in order to build mathematical models of genetic regulatory networks. This approach is based on the mass action law and on the Jacob and Monod operon model. The mathematical models are built symbolically by the *Mathematica* software package *GeneticNetworks*. This package accepts as input the interaction graphs of the transcriptional activators and repressors of a biological process and, as output, gives the mathematical model in the form of a system of ordinary differential equations. All the relevant biological parameters are chosen automatically by the software. Within this framework, we show that concentration dependent threshold effects in biology emerge from the catalytic properties of genes and its associated conservation laws. We apply this methodology to the segment patterning in *Drosophila* early development and we calibrate the genetic transcriptional network responsible for the patterning of the gap gene proteins Hunchback and Knirps, along the antero-posterior axis of the *Drosophila* embryo. In this approach, the zygotically produced proteins Hunchback and Knirps do not diffuse along the antero-posterior axis of the embryo of *Drosophila*, developing a spatial pattern due to concentration dependent thresholds. This shows that patterning at the gap genes stage can be explained by the concentration gradients along the embryo of the transcriptional regulators.

## Introduction

A genetic regulatory network is an ensemble of interactions in a biological process involving proteins, genes and mRNAs. The interactions between different proteins and genes can be done by transcriptional activation and repression at the level of the genes, by protein-protein interactions, and by protein-mRNA interactions.

A genetic regulatory networks is described by a graph where vertices represent genes, proteins, enzymes or other chemical substances. The edges represent transformations, *e. g.*, phosphorylation and dephosphorylation, or activation and inhibitory actions through transcription regulators.

**Figure 1:**
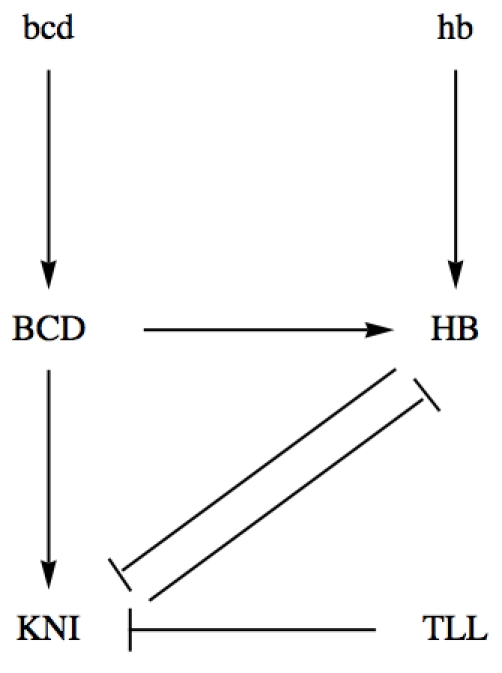
Graph describing the genetic network associated with the production of proteins Bicoid (BCD), Hunchback (HB), Knirps (KNI) and Tailless (TLL) in *Drosophila* early development. mRNAs *hunchback* and *bicoid* are represented by hb and bcd respectively. Arrows represent activations and are listed in the set of ordered pairs 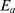. Lines with perpendicular endings represent repressions and are listed in the set 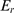.

More precisely, a genetic regulatory networks is described by a double graph 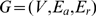, where 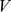 is the set of vertices or nodes of the graph and 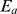 and 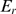 are two sets of ordered pairs of vertices of the double graph. Each ordered pair of vertices defines the activation or the repression mechanism of the first node over the second. In classical graph theory, 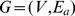 and 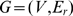 are two graphs with a common set of vertices. For example, in Figure 1, we show the graph of a genetic network associated with the production of the proteins Bicoid (BCD), Hunchback (HB), Knirps (KNI) and Tailless (TLL) in *Drosophila* early development, [1], [2]. In this example, we have 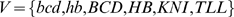, 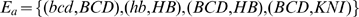 and 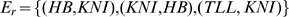.

The graph of Figure 1 has a clear biological meaning. It expresses the fact that BCD is a transcriptional activator of both HB and KNI, HB and KNI proteins both repress each other, and TLL is a repressor of KNI. The vertices of the graph of Figure 1 can represent mRNAs, as in the case of hb and bcd, or proteins, as in the case of BCD and TLL, or genes and proteins simultaneously, as in the case of HB and KNI.

Here we propose a set of rules in order to construct the model equations associated with a genetic regulatory networks described by a double graph 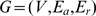. This paper is an attempt to delineate a methodological approach for the construction of mathematical models of gene expression regulation from the principles of chemical kinetics and chemical bound. In the literature, it is often found examples of mathematical models of biological systems described by different sets of equations and characterized by different sets of parameters that are difficult to interpret and to measure experimentally. Making qualitative predictions with these different models has a limited predictable value. For a review on the different approaches see, for example, [3], [4].

In the construction of models for generic regulatory networks, we assume that models can be built with rate equations reflecting a mean field view of the stochastic random motion occurring at the molecular scale. This mean field approach, also called mass action law, is derived from the probabilistic collision laws occurring at the molecular scale. The models originated from this view are described by ordinary differential equations with polynomial vector fields, [5]–[7].

One of the advantages of the mass action law approach is that the mean field rate equations have a direct microscopic interpretation, being associated with the collision mechanism that are in the origin of every reactive process. For model refinement, fluctuations can also be studied through the corresponding master equation. From the experimental point of view, microbiology techniques are strongly anchored in the mass action law or mean field approach, [5], [8].

For genetic regulatory networks described by graphs with a large number of vertices, and a complex structure of edges, the rate equations describing the evolution in time of concentrations are in general difficult to build, and are critically dependent on the assumptions done about the biological and the chemical interactions involved. During the development of these complex models, it is often necessary to test different graph configurations, and to change parameters and initial conditions. Writing by hand all this information is both time-consuming and error-prone.

In order to perform these tasks automatically, we have developed two *Mathematica* software packages, *Kinetics* and *GeneticNetworks*, that execute the symbolic computations associated with the construction of the model equations for a genetic regulatory network. The result of the analysis is in symbolic form, and can be used in *Mathematica*, C or any other simulation software for further numerical integration and graphical analysis. The software packages *GeneticNetworks* and *Kinetics* are freely distributed, [9].

The *Kinetics* package implements the mass action law in its polynomial exact form, computing symbolically the associated rate equations and conservation laws. The parameters generated within *Kinetics* are chemical rate constants.

The package *GeneticNetworks* implements a particular model for protein-gene regulation. This model for the protein-gene regulation is based on the operon model of Jacob and Monod [10] in prokaryotes, and its basic properties have been previously introduced in [11]. The tools in the *GeneticNetworks* software package implement a simplified view of the Molecular Biology Dogma, [12], for protein encoding, translation and transcription, and is consistent with the mass action law. For eukaryotes, transcriptional regulation in is a much more complex issue, involving many redundant binding sites dispersed along genomic sequences. Therefore, in this case, the modeling approach proposed here should be understood as a descriptive approximation to the not well understood eukaryotic regulation mechanisms.

In order to obtain a dimensional reduction on the number of variables in the equations obtained by *Kinetics* and *GeneticNetworks*, it is possible to construct, by a steady state approximation, Hill's function models, [4], [8], [13].

The advantages of using the *Mathematica* computing environment are (i) the possibility of obtaining an exact form for the model equations; (ii) to perform, if necessary, further symbolic simplifications on the models; (iii) to modify the initial theoretical assumptions of the model without having to re-introduce or choose new parameter values; (iv) to make the numerical and graphical analysis of models within the same computing environment; (v) to use of a natural language without a deep knowledge of programming; (vi) to use an easy interface for other programming environments.

To build a model of given genetic regulatory network, the only necessary input to *GeneticNetworks* is the activation and inhibition relationship between genes, mRNAs and proteins. This input is given in the form of the two order pairs of vertices 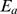 and 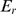 of the network graph. Then, the generation of model equations is done with the *GeneticNetworks* commands. The model equations can be analysed within the *Mathematica* environment or introduced in other programming environments as COPASI, [14], and PottersWhell, [15], for simulation and parameter estimation. These programs are powerful general propose tools in order to numerically simulate solutions of ordinary differential equation and to simulate stochastic models for system biology. At the time of writing this paper, in the site of the Systems Biology Markup Language, http://sbml.org, there were more than 180 registered systems biology simulation programs.

This paper is organized as follows. In the Methods subsection, we briefly review the mass action law of chemical kinetics and we introduce the collision graphs associated with the mass action law. We derive the basic mass action rate equations. A special emphasis is done on mass action conservation laws, an important feature that is in the very foundations of threshold effects in biology. In other approaches, threshold do not result as emergent phenomena, but must be imposed through *ad hoc* regulatory functions (see for example [3] or [4, pp. 237]). We describe the mechanism of genetic regulation based on the Jacob and Monod operon model, [10], and we introduce the modeling assumptions for the construction of the mathematical models of genetic networks described by double graphs. Finally, we give an overview of the *GeneticNetworks* software package.

In the Results section, we show three different applications of the quantitative approach developed here. In the first application, we show, with a very simple example of auto-regulation, that the conservation law constant is a bifurcation parameter for the regulation model, inducing a concentration dependent threshold effect in the model for the production of proteins. This solves the problem of the introduction of *ad hoc* threshold effects in biological simulations, [16]. In the second application, we give a genetic regulatory network inducing a localized spiky pattern along a spatial domain. In this case, the spatial spiky patterns appears without the necessity of other transport mechanisms, as diffusion or advection, but is a consequence of the concentration dependent threshold effect. In the third example, we analyze the experimental data associated with the KNI and HB inhibitory cross regulation in *Drosophila* early development, described by the double graph of Figure 1, and we calibrate this model with the experimental data, without the need of a diffusion hypothesis for the zygotically produced proteins HB and KNI. In the final section, we summarize and discuss the main biological conclusions of the paper.

## Methods

### The mass action law framework of chemical kinetics

In general, an ensemble of chemical reactions is represented by the following collision diagram,
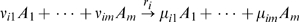
(1)where 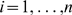. The 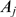, for 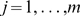, represent chemical substances, as for example, 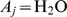. The constants 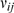 and 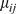 are the stoichiometric coefficients, in general, non-negative integers, and the constants 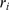 are the rate constants. If 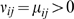, the corresponding substance 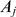 is a catalyst and, if 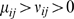, 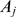 is an autocatalyst. In the diagram (1), there are 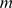 chemical substances and 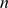 rate constants or chemical reactions.

Under the hypothesis of homogeneity of the solution where reactions occur, the mass action law asserts that the time evolution of the concentrations of the chemical substances is described by the system of ordinary differential equations,
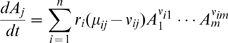
(2)where 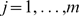, and we use the same symbol to represent both the chemical substance and its concentration. The rate equations (2) are derived under the following assumptions: (i) chemical reactions, when they occur, are due to elastic collisions between the reactants, (ii) homogeneity of the reacting substances in the solution, and (iii) thermal equilibrium of the solution. All the kinetics aspects related with the dependence of the velocity of the reactions on the temperature or pressure are contained in the rate constants 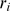. For details see [5].

At the atomic and molecular scale, chemical reactions between molecules can occur only if molecules collide or approach each other to small distances where bounding forces become meaningful. These chemical bounding forces are of electrical or quantum origin, and at distances larger than the mean free path they become less important when compared with the kinetics associated with the molecular motion. As chemical reactions only occur if the chemical substances involved collide, the vector fields associated with the right hand side of (2) are in general quadratic, representing binary collisions. Higher order polynomial vector fields are possible but, at the microscopic level, they are associated to triple or higher order collisions, a situations that occurs with a very low probability. Therefore, we will restrict our examples to models with two-body collisions.

The equations (2) can also be written in the matrix form,
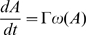
(3)where 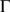 is a 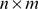 matrix, 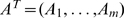, and,
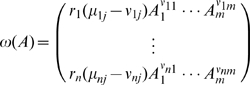
In general, 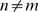, and the equations in system (2) are not all independent. Let us denote by 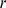 the rank of the matrix 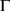. The dimension of the null space of 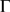 relates with its rank by, 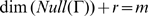 (number of rows of 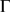). Let 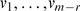 be a basis of the null space of 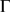, then, 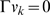, for 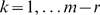. So, by (3), we have,
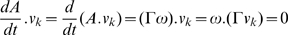
(4)Hence, associated with the differential equations (2), we have the conservation laws,
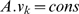
(5)where, 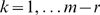.

The *Mathematica* software package *Kinetics* calculates the rate equations (2) describing the time evolution of the concentrations of the substances involved in the reactions described by the collision diagram (1). The package calculates also the corresponding conservations laws (5).

The input of the package is the ensemble of chemical reactions, and the output of the package is the set of differential equations derived by the mass action law. Then, the output can be later analyzed and studied by the analytical and numerical tools in the software package *Mathematica*. In order to avoid long development times, the names of the rate constants are chosen automatically by the program.

The package *Kinetics* has the usual help commands, and we provide the *Mathematica* notebook *KineticsTest.nb* with several self-explanatory examples and computations, [9].

For example, let us describe now a simple protein production model with *Kinetics*. The Molecular Biology Dogma asserts that genes are the templates for protein production, and the standard mechanism for protein production can be represented by the collision diagrams,
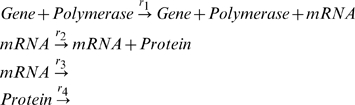
(6)Using the symbols 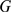, 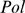, 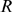 and 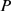 to represent gene, polymerase, mRNA and protein concentrations, respectively, the collision equations (6) are the input for *Kinetics*, with the syntax,
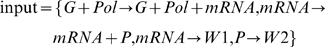
where 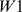 and 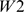 are waist products.

For the collision mechanism (6), the rate equations for the protein concentration, and calculated by the package *Kinetics*, are,
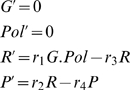
(7)and the rate constants have been chosen automatically by the software package. In this model, genes, polymerase and mRNAs are catalysts, and these equations have the exact solutions,
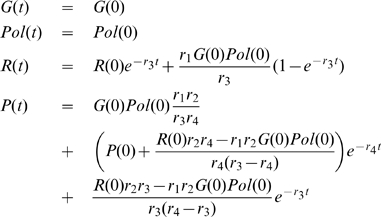
(8)


To simplify the model equations of protein production and maintaining the catalytic properties of genes, in the following, instead of the collision mechanism (6), we use the simplified or reduced kinetic mechanism,
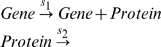
(9)To the reactions (9) correspond the rate equations,
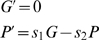
(10)


The rate equations (10) have the solutions,
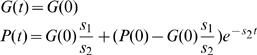
(11)


Comparing the protein solutions in (11) and (8), we conclude that the steady state of the protein in both models is unique and is proportional to the gene concentration. The proportionality constant is different for both models, depending on the rates of the reactions involved. The steady state 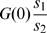 of model (9) has a direct biological meaning: 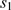 is the rate of protein production and 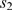 is the rate of protein degradation, and 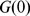 is the initial gene concentration.

In the following, and in order to simplify the description of the transcriptional regulation of genes, we will adopt the mechanism (9) to describe the associated protein production.

In both rate equations (10) and (7), the concentration of gene is constant along time, and therefore the gene concentration is a conservation law. In the following, we will show that the linear conservation laws of the form (5), will have an important role in the determination of steady states and in bifurcations associated with threshold effects.

### A mass action framework to describe genetic regulatory networks based on the protein-gene interaction

In the previous section, we have described a basic model model for the production of proteins, in the framework of the mass action law. Based on this framework, we generalize this view in order to include the case of transcriptional regulation of genes by proteins.

**Figure 2:**
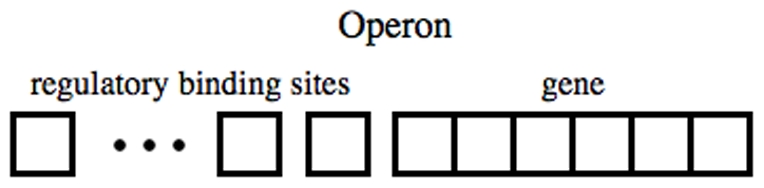
Jacob and Monod operon model for the regulation of protein production. The transcription is regulated by the activators and the repressors binding to the binding sites of the gene.

In order to keep general the approach presented here and to maintain the biological reality of model parameters, we make the following basic modeling assumptions:

1. In order to describe quantitatively the protein production (concentration) within the Molecular Biology Dogma, we only consider genes and proteins. Intermediate substances in the regulatory mechanism like catalysts, polymerases and mRNAs are not considered. A model of protein production has been presented and analyzed at the end of the previous section.
2. The regulation of protein production by the template gene is based on the Jacob and Monod operon model, [10]. Namely, every gene has associated a certain number of binding sites where transcription factors can bind — activators or repressors, Figure 2. The regulation of activations and repressions occurs only through the binding sites. For a given double graph of interactions, the number of binding sites of a gene is determined by the number of edges that end up in the corresponding graph node.
3. Transcription factors are the proteins associated with the vertices that activate or inhibit the production of other proteins. The vertex of a graph represent a transcription factor only if it is the initial point of a edge of activation or inhibition. If a vertex has incoming and outgoing edges of any type, this vertex represents symbolically a protein and a gene with several binding states.
4. Each transcription factor has its own binding site in the gene strand, or each gene has only one binding site for all the regulators. Both cases are treated separately in the model. We assume that when at least one activator is bound to a gene, the transcription is activated with a particular production rate for each combinatorial possibility of all the remaining binding sites.

For example, in the double graph of the biological mechanism of Figure 1, we have the following chemical substances,
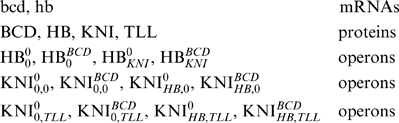
(12)The description of the time evolution of protein concentrations of the mechanisms of Figure 1 involves one rate equation for each substance in (12), except eventually for bcd and hb. As proteins are produced from a gene template, the symbol associated to each vertex of the graph represents a protein. The operon states are represented by the same symbol with superscripts and lowerscripts. The superscripts positions indicate the binding or unbinding of transcriptional activators. The lowerscripts positions indicate the binding or unbinding of transcriptional repressors. In the *GeneticNetworks* software package, bcd, hb, BCD, HB, KNI and TLL are the names of the vertices of the regulation graphs, but the operon variables in (12) are generated by the software.

The model associated with a given double graph contains the rate equations for the proteins and the operons in its different states. We also assume by default that proteins always degrade and genes are autocatalytic substances that never degrade. The first assumption implies that protein concentrations remain bounded in time, and the second assumption implies the existence of a conservation law for the concentration of the operon states. Using the symbolic tools of *Mathematica*, other assumptions can be introduced at this stage of model construction.

### The *GeneticNetworks* software package

*GeneticNetworks* is a software package that generates the rate equations for the concentrations of genes and proteins in a regulatory network, [9]. The starting point is the double graph of activations and repressions, 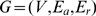. The inputs for *GeneticNetworks* are two strings, *activation* and *repression*, that describe the transcriptional activations and repressions of proteins on genes. In the graph, the same symbol is used to denote both a gene and the corresponding produced protein. As we have seen in (12), the set of variables for the regulation model is constructed with the vertex symbols.

**Figure 3:**
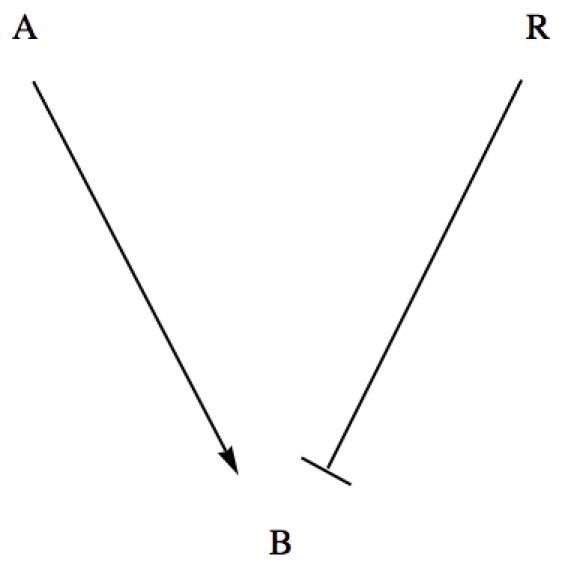
Double graph associated with the input strings (13) for the *GeneticNetworks* software package.

For example, using as input for *GeneticNetworks* the interaction strings,
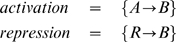
(13)the double graph of the genetic network (13) is shown in Figure 3. In this case, the double graph 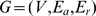 is characterized by the sets, 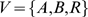, 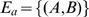 and 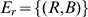.

In the interaction mechanism (13), protein A activates gene B, and protein R represses gene B. Therefore, the variables of the mechanism (13) are,
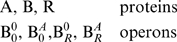
(14)


The following functions are defined in the *GeneticNetworks* package:

- NetworkGraph, ManipulateGraph
- Reactions, ReactionsOneSite, ReactionGraph
- SubstanceNames, SubstanceVariables, SubstanceInitialConditions
- ParameterNames, ParameterInput
- Equations
- ConservationLaws

With these functions, we calculate the model equations associated with the input strings (13), calculate automatically the number of variables of the model, define all the relevant parameters and calculate the rate equations. For example, to the genetic regulatory network (13), the *GeneticNetworks* package builds the mass action law type collision diagrams,
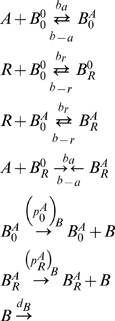
(15)To these collision diagrams, we have the mass action law rate equations,
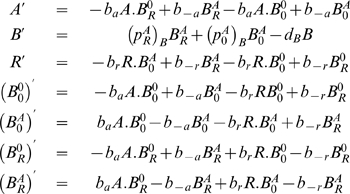
(16)and the conservation law,
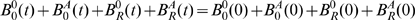
(17)From the conservation law (17), we can eliminate one of the equations in (16). In this genetic network, we have assumed that the protein concentrations of 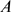 and 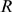 are constant along time.

The rate equations (16) define a mass action law based model for the genetic regulatory network of Figure 3.

In the implementation of *GeneticNetworks*, we have two possible modeling choices. In one choice, each different regulator has its own binding site, and the model diagrams (15) have been constructed with this assumption. For the second choice, we consider that there is only one binding site in the operon where all the regulators bind. In this case, the collision diagrams associated with the genetic network (13) and calculated in the *GeneticNetworks* package are,
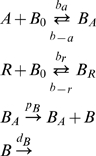
(18)By the mass action law, the ReactionsOneSite command leads to the rate equations,
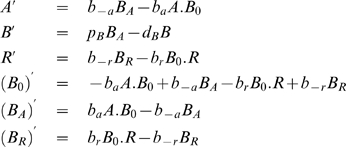
(19)The equations (19) have the conservation law,
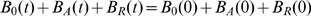
(20)


The two models (15) and (18) for the genetic network (13) are different and these two choices are implemented in the *GeneticNetworks* package. For the dynamical analysis of a particular case of the distinction between the two models (15) and (18), see [11]. Below, we will show with a specific example that these two different regulation choices lead to qualitatively and quantitatively similar results.

## Results

### An emerging concentration threshold in the dynamics of a self-activating protein

**Figure 4:**
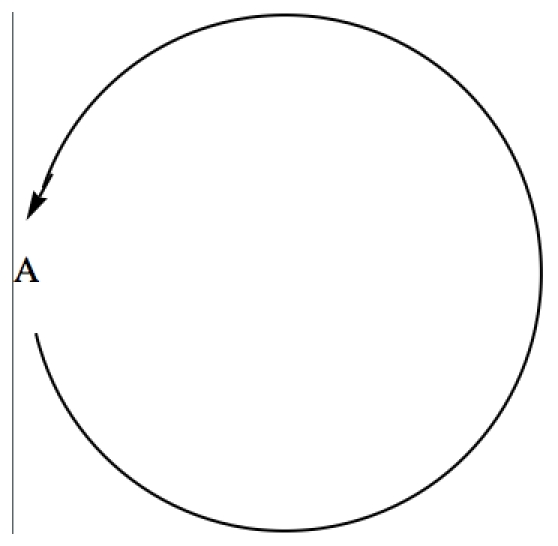
Regulation graph describing a self-activating protein.

As an application of the rules describing a genetic regulatory network just introduced, we discuss now the basic role of the conservation laws in the occurrence of threshold effects in regulation mechanisms. We study the case of a self-activating protein, where the produced protein activates its own production, Figure 4.

The simplest self-activating genetic network is described by the input tables,
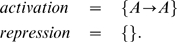
The reactions and the parameters involved in this activation can be obtained by the *GeneticNetworks* command Reactions, followed by the command ReactionGraph,
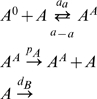
(21)where 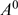 and 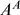 are the operon states and 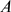 is the corresponding protein.

With the command Equations, we get the rate equations,
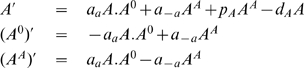
(22)Finally, with the command ConservationLaws, we find,
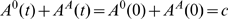
(23)where 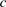 is a constant.

Introducing (23) into (22), the independent set of rate equations describing the process (21) is,
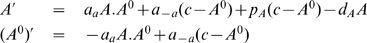
(24)


We analyze now the steady state and the phase space structure of the solutions of equations (24). Equations (24) have two steady states with coordinates,
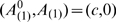
and,
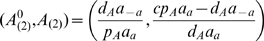
As the coordinate of the two steady sates are dependent of 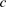, by (23), the steady state coordinates are dependent of the initial concentrations of the operon.

Let 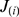, 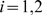 be the Jacobian of equation (24) evaluated at the fixed points. As,
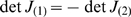
and,
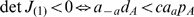
then, we have,
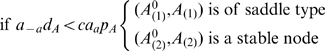
(25)

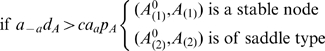
(26)As, for 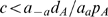, 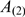 is negative, the protein concentration at the steady state of the rate equations (24) is zero (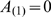). For 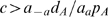, the protein concentration at equilibrium is 
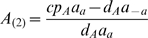
. Therefore, the conservation law (23) tune a bifurcation for 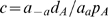 (transcritical bifurcation), implying the existence of a threshold effect tuned by the conservation law parameter 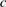.

**Figure 5:**
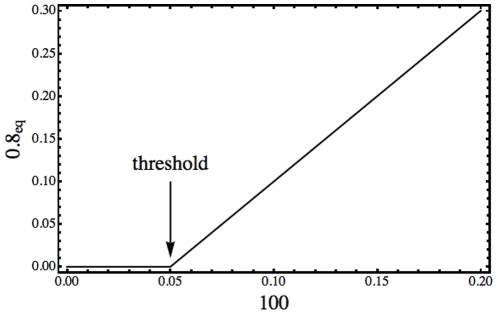
Dependence of the protein steady state on the total concentration of the gene. Below the bifurcation or threshold 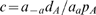, the equilibrium value of protein concentration 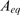 is zero, while above it takes the value 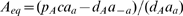. The parameters are: 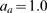, 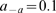, 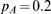 and 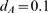.

In Figure 5, we show the dependence of the protein steady state on the total concentration of the gene. In this simple regulation model, where both protein and gene concentrations are modeled, the steady state of the protein depends on the initial concentration of the corresponding operon states. On the other hand, the initial concentration of the operon states induce a bifurcation from a quiescent state to a non zero steady state. This is a threshold effect that emerges from the dynamics (24). As the steady state protein concentration depend on 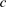, the “after threshold” concentration values depends continuously on the operon initial concentration 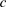. In the following, we will see that in networks with more than one node, the steady state depends also on the concentration of other transcriptional regulators, and these concentration dependent thresholds can be in the origin of spatial patterning.

### Spatial distribution and steady states

We have focused on genetic regulatory models without specifying a spatial localization. In genetic networks describing some biological process, the initial concentration of proteins can significantly vary across tissues. For example, in some developmental processes, proteins show a non-uniform concentration along embryos, with very sharp slopes, playing a basic role in the establishment of body plans of organisms. A well known case is the *Drosophila* segmentation, where variations on protein concentrations across the embryo induces protein patterning, [17]–[19]. One of such genetic regulatory networks is the one represented in Figure 1, [2].

**Figure 6:**
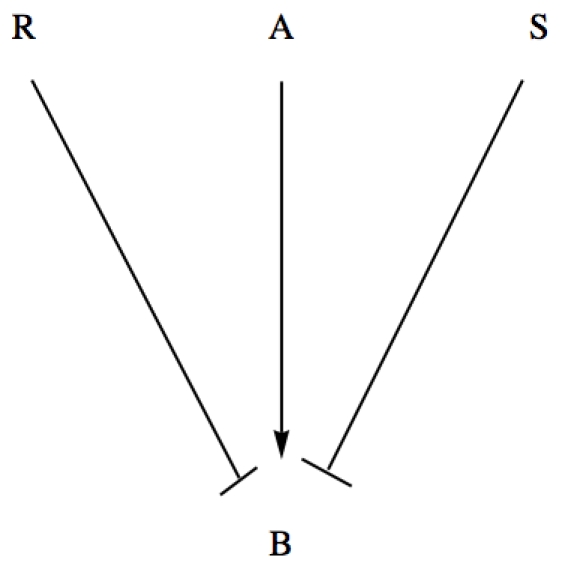
Genetic regulatory network for the production of protein 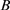 with one activator 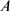 and two repressors 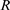 and 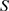.

To show that patterning can be explained by the non homogeneity of initial conditions of regulators across tissues, we analyze a genetic regulatory network for the production of a protein 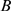, regulated by one activator 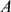 and two repressor proteins 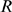 and 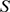, Figure 6. To simplify our analysis, we take the competitive case, where the activator and the repressors bind to the same binding site of the operon of protein 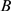.

To simplify further, we assume that the spatial distribution of the proteins 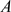, 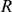 and 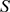 are constant in time. Under these conditions and with the regulation model developed in the package *GeneticNetworks*, we obtain the system of linear rate equations,
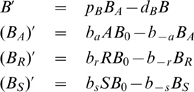
(27)where the derivative is in order to time, 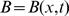, 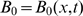, 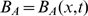, 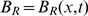, 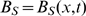, 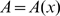, 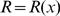 and 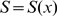. These concentration variables are defined in a spatial one-dimensional bounded region of the real line (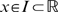). The following conservation law holds,
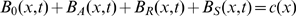
where 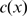 is a constant, depending eventually of the spatial independent coordinate 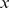. The system of rate equations (27) has one steady state with coordinates,
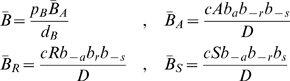
where,
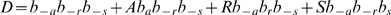
Choosing 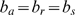, and 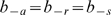, the steady state concentration of the protein 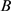, is,
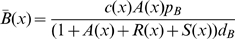
(28)


**Figure 7:**
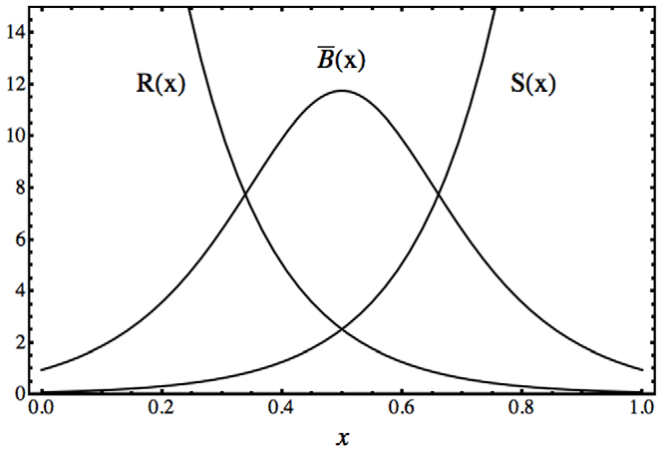
Steady states of proteins 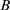, 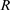 and 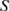 for the genetic regulatory network of Figure 6. The steady state of protein 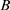 shows a spiky profile, resulting from the inhibitory action of proteins 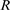 and 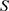. In this model, we have considered that the concentrations of 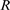 and 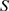 are constant in time and non-homogeneous in space. The activator protein 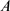 has been considered constant along the spatial region.

In Figure 7, we show the steady state concentration (28) of protein 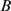, as a function of a spatial coordinate, 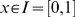. We have considered the initial distributions 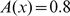, 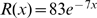, 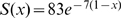, 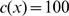, and the parameter value 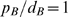. In this case, due to the inhibitory regulation of the repressor proteins 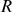 and 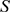, the steady distribution of protein 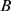 is spiky. We have analyzed the same genetic network of Figure 6 with a model with different binding sites for each regulator. The final result is similar with the one shown in Figure 7. This shows that the two model approaches in *GeneticNetworks*, with one binding site and with several binding sites in the operon, give similar qualitative results. When, the calibration and validation of models is not a problem, we can use the simplest one binding site regulation model in order to describe a given genetic regulatory network. The one-regulator site framework leads to simpler mathematical models.

The solution (28) shows that steady states can depend on the initial conditions of the regulators (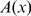, 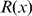 and 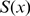) and on the initial conditions of the catalytic agents (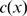), showing that spatial patterning can be a direct consequence of the non homogeneity of initial conditions.

In this model, we have considered that the concentrations of 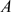, 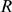 and 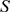 are constants, implying that the model equations (27) describe a system where 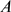, 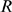 and 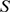 have a fast recovery time. This situation only occurs in thermodynamically open systems, as is the case of biological systems.

### Cross-regulation in *Drosophila*

**Figure 8:**
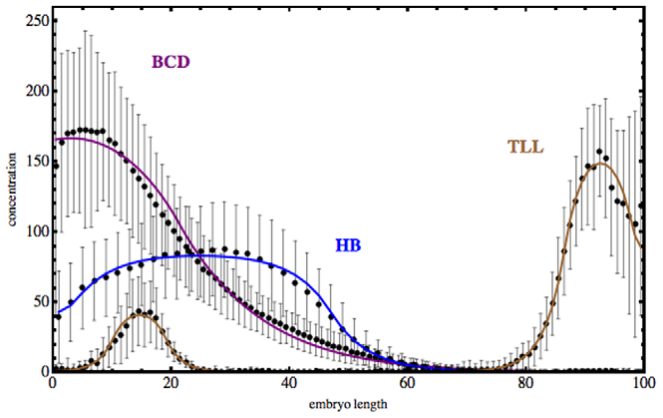
Concentration of protein Hunchback (HB) at the end of cleavage cycle 13, and of Bicoid (BCD) and Tailless (TLL) proteins at the cleavage cycle 14, along the antero-posterior axis of the embryo of *Drosophila*. The embryo length has been scaled from 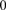 to 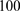, and the units in the vertical axis are proportional to protein concentration. The data has been taken from the FlyEx database. The continuous curves represent the fitted mean distribution of the concentration of proteins calculated from the data of 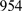 embryos. These curves are the initial conditions for a model obtained with the software package *GeneticNetworks* for the production of the proteins KNI and zygotically produced HB, during cleavage cycle 14.

In *Drosophila* early development, some proteins as Bicoid (BCD) and Hunchback (HB) are translated from mRNA of maternal origin. Early in the first developmental stages of *Drosophila*, at cleavage stage 13, BCD and HB proteins form a stable concentration gradient along the antero-posterior axis of the *Drosophila* embryo. In Figure 8, we show the data for these protein gradients, taken from the FlyEx database, [[20], [21], http://flyex.ams.sunysb.edu/flyex/]. These stable gradients have are established by diffusion processes occurring in the syncytial blastoderm of the embryo, [17]–[19], [22]. At a latter stage, in the 14th cleavage stage, other proteins as Knirps (KNI) show segments characterized by spiky concentration patterns along the antero-posterior axis of the embryo, [1], [2], [23]–[26].

We show now that the patterning of HB and KNI proteins as observed at late cleavage stage 14 of the embryo of *Drosophila* is due to the concentration gradients of proteins at an early developmental stage. This results follows from the concentration dependent threshold effect, just described previously, without assuming diffusion for KNI and for zygotically produced HB. For that, we have taken the genetic regulatory network of Figure 1, describing the genetic regulation of HB and KNI, and we have used the package *GeneticNetworks* to describe the production of proteins Hunchback and Knirps during the cleavage cycles 14 of the developing embryo of *Drosophila*.

Hunchback and Knirps proteins are both activated by the maternally produced protein Bicoid, Figure 1, and they mutually repress each other. The protein Knirps is also repressed by the protein Tailless. Therefore, the genetic regulatory network model obtained with the package *GeneticNetworks* should lead to the experimental profiles of HB and KNI, as observed at cleavage cycle 14. As the model obtained with the *GeneticNetworks* package is a system of ordinary differential equations that depend on initial data, we have assumed that, at the end of cleavage cycle 13 and beginning of the 14th, the proteins BCD, TLL and HB of maternal origin have a non homogeneous distribution along the embryo, as shown in Figure 8.

**Figure 9:**
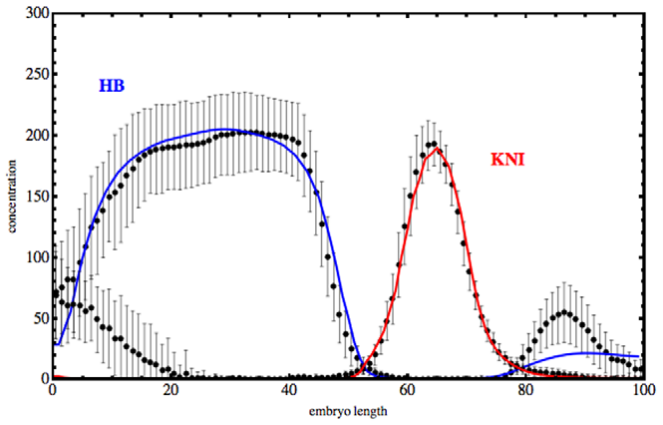
The dots with error bars are the mean experimental profiles of the proteins HB and KNI at cleavage cycle 14, taken along the antero-posterior axis of the embryo, for several *Drosophila* embryos. The mean distribution of the concentration of proteins has been calculated from the data of 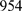 embryos, taken from the FlyEx database. The embryo length has been scaled from 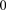 to 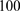, and the units in the vertical axis are proportional to protein concentration. The continuous curves show the predictions of the model of production of proteins HB and KNI. The model equations have been obtained with the software package *GeneticNetworks*.

In Figure 9, we show the experimental profiles of HB and KNI proteins at cleavage cycle 14, as well as a fit of the experimental data obtained with the model built with the software package *GeneticNetworks*. The model equations is a system of 14 ordinary differential equations for the proteins and corresponding operon states, and have 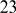 free parameters. In the ordinary differential equations of the model, we have considered that time is also a free parameter. The protein profiles shown in Figure 9 are out of equilibrium patterns, obtained with the integration time 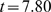 s. These equations and the fitted parameter values are presented in Text S1.

To fit the experimental data with the mathematical model, we have assumed that the initial protein concentrations of BCD and TLL are constant over time, and we have also assumed that each regulator has its own binding site. To find the numerical values of the model parameters in order to calibrate the model, we have used an optimization technique based on a genetic evolutionary algorithm, minimizing a sum of chi square functions, [23], [27].

The fitted curves in Figure 9 show a very good agreement with the experimental data. HB fits well in the embryo length range 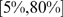, and KNI fits well in the embryo length range 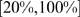. The quality of the fits has been measured by the penalized chi square test. For the two fits in Figure 9, we have obtained the reduced chi square values 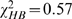 and 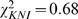. This calibration of the genetic regulatory network of Figure 1 suggests that the anterior and posterior regions of the embryo are under the control of additional regulators. This same conclusion has been obtained in [25], but within a different regulation model. On the other hand, this mass action law model without diffusion at the level of gap genes is simpler than other full diffusion models, and is consistent with the reverse engineering methodology described in [26].

## Discussion

We have presented a software tool to build mathematical models of genetic regulatory networks. The input of the package is the graph containing the list of transcriptional activators and repressors of the network. The software implements an approach based on the mass action law and on the operon regulation model in prokaryotes. We have also assumed in general that genes are catalytic substances presented in any genetically controlled biological process. For eukaryotic organisms, the modeling approach proposed here should be understood as a descriptive approximation to the not well understood eukaryotic regulation mechanisms.

Within this approach, the usual threshold concept in biology emerges as a bifurcation phenomenon of the model equations. These bifurcations are tuned by the conservation law constants of the equations, resulting from the catalytic role of genes. This corroborates the view that threshold effects should be anchored on bifurcation phenomena, [16].

Another consequence of the modeling approach presented here is that positional information in developmental processes can be described by the non-homogeneity in the spatial distribution of the concentration of regulators, and is not necessarily associated with other physical processes of transport or diffusion. Other models for *Drosophila* development include a balance between protein diffusion and degradation, [2], [28]–[30]. Recently, some criticism to the diffusion-degradation hypothesis for proteins, [31], suggest that it is important to search for other mechanisms of pattern formation, [22], [32], [33]. The results presented here show that other mechanisms of spatial patterning are possible.

In conclusion, we have calibrated a genetic regulatory network for the production of Hunchback and KNI during the 14th cleavage stage of the embryo of *Drosophila*, without assuming the hypothesis of protein diffusion for KNI and zygotically produced HB, and we have presented evidence that gap gene protein segments are out of equilibrium patterns. The genetic regulatory network of Figure 1 describes well the gap gene protein concentration of HB in the embryo length region 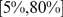, as well as the gap gene protein concentration of KNI in the embryo length region 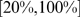. The out of equilibrium pattern hypothesis has been suggested in [28] in the framework of a model assuming that the HB and KNI proteins diffuse along the antero-posterior axis of the embryo of *Drosophila*. In [30], patterning at the gap gene stage is associated with the existence of attractors in an high dimensional phase-space, implying that gap gene patterns are obtained as steady state patterns. The necessity of concentration dependent thresholds in the gap-gene *Drosophila* patterning has been discussed in [24], and modeled through a Hill type response function with diffusion. Here, with mass action law approach, gap-gene patterns emerge from the concentration dependent thresholds that result from the catalytic role of genes in organisms.

## Supporting Information

Text S1 Ordinary differential equation model describing the genetic regulatory network of Figure 1.
(0.06 MB PDF)
