# Supplementary material for: A Software Tool to Model Genetic Regulatory Networks. Applications to the Modeling of Threshold Phenomena and of Spatial Patterning in Drosophila
Source: PLoS One. 2010 May 27;5(5):e10743. doi: 10.1371/journal.pone.0010743 (PMC2877713; doi:10.1371/journal.pone.0010743)
Supplement: Text S1 — Ordinary differential equation model describing the genetic regulatory network of Figure 1. (0.06 MB PDF) [file pone.0010743.s001.pdf]

Supporting Information S1

System of ordinary differential equations of the genetic regulatory network of Figure 1, obtained with the *GeneticNetworks* software package.

$$
\begin{aligned}
HB'[t] &= -HB[t]d_{HB} + \left(p_0^{BCD\$}\right)_{HB} HB_0^{BCD\$}[t] + \left(p_{KNI}^{BCD\$}\right)_{HB} HB_{KNI}^{BCD\$}[t] \\
&- HB[t]kni_{hb}KNI^0_{0,0}[t] - HB[t]kni_{hb}KNI^{BCD\$}_{0,0}[t] \\
&- HB[t]kni_{hb}KNI^0_{0,TLL\$}[t] - HB[t]kni_{hb}KNI^{BCD\$}_{0,TLL\$}[t] \\
&+ kni_{-hb}KNI^0_{HB,0}[t] + kni_{-hb}KNI^{BCD\$}_{HB,0}[t] + kni_{-hb}KNI^0_{HB,TLL\$}[t] \\
&+ kni_{-hb}KNI^{BCD\$}_{HB,TLL\$}[t] \\
KNI'[t] &= -KNI[t]d_{KNI} - KNI[t]hb_{kni}HB^0_0[t] - KNI[t]hb_{kni}HB_0^{BCD\$}[t] \\
&+ hb_{-kni}HB^0_{KNI}[t] + hb_{-kni}HB^{BCD\$}_{KNI}[t] + \left(p_{0,0}^{BCD\$}\right)_{KNI}KNI^{BCD\$}_{0,0}[t] \\
&+ \left(p_{0,TLL\$}^{BCD\$}\right)_{KNI}KNI^{BCD\$}_{0,TLL\$}[t] + \left(p_{HB,0}^{BCD\$}\right)_{KNI}KNI^{BCD\$}_{HB,0}[t] \\
&+ \left(p_{HB,TLL\$}^{BCD\$}\right)_{KNI}KNI^{BCD\$}_{HB,TLL\$}[t] \\
\left(HB^0_0\right)'[t] &= -BCD\$hb_{bcd\$}HB^0_0[t] - KNI[t]hb_{kni}HB^0_0[t] \\
&+ hb_{-bcd\$}HB_0^{BCD\$}[t] + hb_{-kni}HB^0_{KNI}[t] \\
\left(HB_0^{BCD\$}\right)'[t] &= BCD\$hb_{bcd\$}HB^0_0[t] - hb_{-bcd\$}HB_0^{BCD\$}[t] \\
&- KNI[t]hb_{kni}HB_0^{BCD\$}[t] + hb_{-kni}HB^{BCD\$}_{KNI}[t] \\
\left(HB^0_{KNI}\right)'[t] &= KNI[t]hb_{kni}HB^0_0[t] - BCD\$hb_{bcd\$}HB^0_{KNI}[t] \\
&- hb_{-kni}HB^0_{KNI}[t] + hb_{-bcd\$}HB^{BCD\$}_{KNI}[t] \\
\left(HB^{BCD\$}_{KNI}\right)'[t] &= KNI[t]hb_{kni}HB_0^{BCD\$}[t] + BCD\$hb_{bcd\$}HB^0_{KNI}[t] \\
&- hb_{-bcd\$}HB^{BCD\$}_{KNI}[t] - hb_{-kni}HB^{BCD\$}_{KNI}[t] \\
\left(KNI^0_{0,0}\right)'[t] &= -BCD\$kni_{bcd\$}KNI^0_{0,0}[t] - HB[t]kni_{hb}KNI^0_{0,0}[t] \\
&- TLL\$kni_{tll\$}KNI^0_{0,0}[t] + kni_{-bcd\$}KNI^{BCD\$}_{0,0}[t] \\
&+ kni_{-tll\$}KNI^0_{0,TLL\$}[t] + kni_{-hb}KNI^0_{HB,0}[t] \\
\left(KNI^{BCD\$}_{0,0}\right)'[t] &= BCD\$kni_{bcd\$}KNI^0_{0,0}[t] - kni_{-bcd\$}KNI^{BCD\$}_{0,0}[t] \\
&- HB[t]kni_{hb}KNI^{BCD\$}_{0,0}[t] \\
&- TLL\$kni_{tll\$}KNI^{BCD\$}_{0,0}[t] + kni_{-tll\$}KNI^{BCD\$}_{0,TLL\$}[t] \\
&+ kni_{-hb}KNI^{BCD\$}_{HB,0}[t] \\
\left(KNI^0_{0,TLL\$}\right)'[t] &= TLL\$kni_{tll\$}KNI^0_{0,0}[t] - BCD\$kni_{bcd\$}KNI^0_{0,TLL\$}[t] \\
&- HB[t]kni_{hb}KNI^0_{0,TLL\$}[t] - kni_{-tll\$}KNI^0_{0,TLL\$}[t] \\
&+ kni_{-bcd\$}KNI^{BCD\$}_{0,TLL\$}[t] + kni_{-hb}KNI^0_{HB,TLL\$}[t] \\
\left(KNI^{BCD\$}_{0,TLL\$}\right)'[t] &= TLL\$kni_{tll\$}KNI^{BCD\$}_{0,0}[t] + BCD\$kni_{bcd\$}KNI^0_{0,TLL\$}[t] \\
&- kni_{-bcd\$}KNI^{BCD\$}_{0,TLL\$}[t] - HB[t]kni_{hb}KNI^{BCD\$}_{0,TLL\$}[t] \\
&- kni_{-tll\$}KNI^{BCD\$}_{0,TLL\$}[t] + kni_{-hb}KNI^{BCD\$}_{HB,TLL\$}[t] \\
\left(KNI^0_{HB,0}\right)'[t] &= HB[t]kni_{hb}KNI^0_{0,0}[t] - BCD\$kni_{bcd\$}KNI^0_{HB,0}[t] \\
&- kni_{-hb}KNI^0_{HB,0}[t] - TLL\$kni_{tll\$}KNI^0_{HB,0}[t] \\
&+ kni_{-bcd\$}KNI^{BCD\$}_{HB,0}[t] + kni_{-tll\$}KNI^0_{HB,TLL\$}[t]
\end{aligned}
$$

$$
\begin{aligned}
\left(KNI^{BCD\$}_{HB,0}\right)'[t] &= HB[t]kni_{hb}KNI^{BCD\$}_{0,0}[t] + BCD\$kni_{bcd\$}KNI^{0}_{HB,0}[t] \\
&- kni_{-bcd\$}KNI^{BCD\$}_{HB,0}[t] - kni_{-hb}KNI^{BCD\$}_{HB,0}[t] \\
&- TLL\$kni_{tll\$}KNI^{BCD\$}_{HB,0}[t] + kni_{-tll\$}KNI^{BCD\$}_{HB,TLL\$}[t] \\
\left(KNI^{0}_{HB,TLL\$}\right)'[t] &= HB[t]kni_{hb}KNI^{0}_{0,TLL\$}[t] + TLL\$kni_{tll\$}KNI^{0}_{HB,0}[t] \\
&- BCD\$kni_{bcd\$}KNI^{0}_{HB,TLL\$}[t] - kni_{-hb}KNI^{0}_{HB,TLL\$}[t] \\
&- kni_{-tll\$}KNI^{0}_{HB,TLL\$}[t] + kni_{-bcd\$}KNI^{BCD\$}_{HB,TLL\$}[t] \\
\left(KNI^{BCD\$}_{HB,TLL\$}\right)'[t] &= HB[t]kni_{hb}KNI^{BCD\$}_{0,TLL\$}[t] + TLL\$kni_{tll\$}KNI^{BCD\$}_{HB,0}[t] \\
&+ BCD\$kni_{bcd\$}KNI^{0}_{HB,TLL\$}[t] - kni_{-bcd\$}KNI^{BCD\$}_{HB,TLL\$}[t] \\
&- kni_{-hb}KNI^{BCD\$}_{HB,TLL\$}[t] - kni_{-tll\$}KNI^{BCD\$}_{HB,TLL\$}[t]
\end{aligned}
$$

The BCD(x), TLL(x), with $x \in [0, 100]$, are the distribution of BCD and TLL proteins along the antero-posterior axis of *Drosophila* embryo. Their values are represented in Figure 8. The initial distribution of HB is also represented in Figure 8. The initial concentration of the protein KNI is zero. All the operon states have zero initial conditions, except $\mathrm{KNI}^0_{0,0}$ and HB^0_0 whose initial states are free parameters: $\mathrm{KNI}^0_{0,0}[0] = GC_{KNI}$ and $\mathrm{HB}^0_0[0] = GC_{HB}$.

The parameter values for the fits in Figure 9 are represented in the table below.

$hb_{bcd} = 0.911508$	$hb_{kni} = 1.990365$	$kni_{bcd\$} = 0.694221$
$kni_{hb} = 1.139939$	$kni_{tll} = 1.99477$	$hb_{-bcd\$} = 0.199906$
$hb_{-kni} = 0.050465$	$kni_{-bcd\$} = 0.050144$	$kni_{-hb} = 0.053843$
$kni_{-tll} = 0.118673$	$(p_0^{BCD\$})_{HB} = 15.11278$	$(p_{KNI}^{BCD\$})_{HB} = 0.0$
$(p_{0,0}^{BCD\$})_{KNI} = 16.54227$	$(p_{HB,0}^{BCD\$})_{KNI} = 0.024902$	$(p_{0,TLL\$}^{BCD\$})_{KNI} = 16.16442$
$(p_{HB,TLL\$}^{BCD\$})_{KNI} = 0.021352$	$d_{HB} = 0.900265$	$d_{KNI} = 0.955021$
$Time = 7.796199$	$PC_{HB} = 0.117246$	$PC_{KNI} = 1.912638$
$GC_{HB} = 282.042$	$GC_{KNI} = 299.5284$	

Table 1. Parameter values of the fits shown in Figure 9. The first 18 parameters are the free parameters of the differential equation model shown above. The system of equations has been integrated numerically with an adaptive step size, starting from $\Delta t = 0.001$, and the parameter $Time$ is the total integration time. The parameters PC_{HB} and PC_{KNI} are scale factors that multiply the differential equation solutions $\mathrm{HB}(Time, x)$ and $\mathrm{KNI}(Time, x)$, with $x \in [0, 100]$, in order to avoid biasing of data. Note that experimental data obtained by fluorescent methods indicate a concentration that is proportional to the actual concentration of HB and KNI proteins, and these scaling factors are unknown and are different for each protein. The constants GC_{HB} and GC_{KNI} are the total gene concentrations that are responsible for the transcription and translation of proteins HB and KNI. If the genes encoding HB and KNI were both active in each nucleus, we should have $GC_{HB} = GC_{KNI}$.
